# How many lives are at stake? Assessing 2030 sustainable development goal trajectories for maternal and child health

**DOI:** 10.1136/bmj.k373

**Published:** 2018-02-15

**Authors:** John W McArthur, Krista Rasmussen, Gavin Yamey

**Affiliations:** 1 Global Economy and Development Program, Brookings Institution, Washington, DC 20036, USA; 2 Center for Policy Impact in Global Health, Duke Global Health Institute, Duke University, Durham NC, USA

## Abstract

**John W McArthur**, **Krista Rasmussen**, and **Gavin Yamey** examine how far countries have to go to meet the targets for maternal and child mortality and what needs to be done to help them

The launch of the 17 sustainable development goals in 2016 introduced a new era for the global health and development community. The preceding millennium development goals (MDGs) set targets to reduce the mortality rate for children under 5 years by two thirds and the maternal mortality ratio by three quarters between 1990 and 2015, with special focus on the poorest countries. Overall, the world ended up reducing child mortality by an estimated 55% and maternal mortality ratio by 44%, while countries classified as “least developed” by the United Nations experienced a 60% decline in child mortality and 52% decline in maternal mortality.^1^ ^2^ At least 10.1 million and as many as 19.4 million additional children’s and mothers’ lives are estimated to have been saved compared with pre-MDG trajectories.^3^ Many of the biggest improvements occurred in sub-Saharan Africa.^3^

The new goals, which apply to all countries and run to 2030, include one health goal, SDG 3—to “ensure healthy lives and promote wellbeing for all at all ages”—with 13 associated targets. Target 3.1 calls for the global maternal mortality ratio to be below 70 deaths per 100 000 live births, a 68% reduction in only 15 years. Target 3.2 calls for all countries to lower their child mortality to at most 25 per 1000 live births and their neonatal (age 0-28 days) mortality to at most 12 per 1000 live births. Are countries on course to meet the new targets, and, if not, what do they need to do to accelerate their progress?

## Current prospects for reaching the targets

Recent studies have considered this question with a focus on individual indicators, noting some of the large accelerations required.^4^ ^5^ But it is also important to examine the question from the perspective of individual countries, across targets, to identify where new action needs to be focused.

We examined recent trends in child and maternal mortality and extrapolated them forward to 2030. We first calculated each country’s proportional annualised average rate of fall in these indicators for the most recent 10 years with available data: from 2005 to 2015 for maternal mortality ratio and 2006 to 2016 for child mortality.^1^ ^2^ Next, we extrapolated the 10 year trend out to 2030, assuming no change in the rate of decline (the “business as usual” scenario). Then we used country level birth projections, taken from the UN Population Division’s 2017 population prospects, to estimate birth weighted global child and maternal mortality aggregates, alongside country level trajectories for the absolute number of maternal and child deaths out to 2030.^6^

We have not tried to predict 2030 outcomes but present here trend analysis using best available data. Official mortality estimates for recent years are likely to be updated in future, especially for countries with high mortality. Some researchers have also argued that countries with rapid recent falls in child mortality are likely to experience slower rates of decline in the future.^7^ If this occurs, it would only amplify the estimated 2030 outcome gaps for relevant countries. However, the unprecedented structural shift in global health trajectories since the early 2000s, especially among low income countries, underscores the difficulty of predicting future outcomes.^8^ Our analyses provide estimates of outcomes only if recent reported trajectories continue.

**Fig 1:**
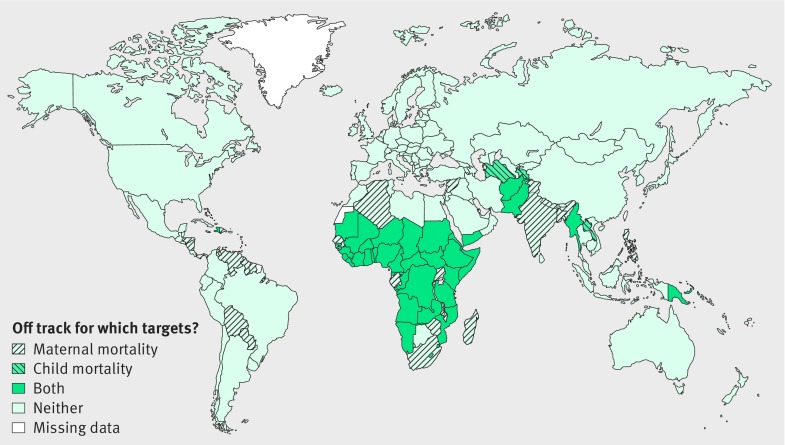
Countries that are off track for 2030 targets for maternal or child mortality^1^ ^2^

Figure 1 summarises the country level results, which are also available in the appendix on bmj.com. Of the 181 countries with data for both indicators, 42 (23%) are off track for both maternal and child mortality; 28 (15%) are off track only for maternal mortality; and six (3%) are off track only for child mortality. Another three small island states (Dominica, Marshall Islands, and Nauru) are off track for child mortality but do not have data available for maternal mortality. Thirty five of the countries that are not on track for either target are in sub-Saharan Africa and had a total population of 820 million in 2015.

## How much acceleration is required?

**Fig 2:**
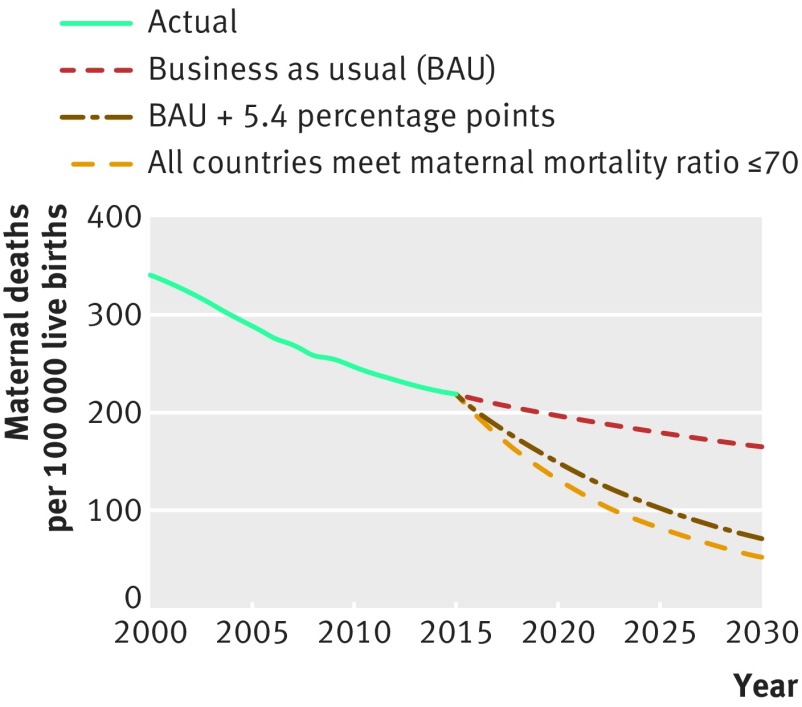
Scenarios for global trajectory for maternal mortality ratio to 2030^2^ ^6^

As shown in figure 2, if “business as usual” trends from 2005 to 2015 continue to 2030, the global maternal mortality ratio will fall only to 164/100 000 live births. This is equivalent to an ongoing overall reduction of just 1.9% a year, slower than the rate of 2.7% per year from 2005 to 2015. The aggregate slowdown occurs because countries with high maternal mortality and slow rates of decline account for a gradually larger share of the world’s projected births over the period.

The 2030 target for maternal mortality can be approached through two pathways. One calculates the aggregate rate of decline required for the world to reach a global maternal mortality ratio of 70/100 000 live births. This works out to a 7.3% annual rate of decline, which could be achieved if each country realised an extra 5.4 percentage point annual acceleration on top of its current “business as usual” trend. As a recent historical reference point, only 11 countries improved their rate of decline by at least 5.4 percentage points between 1990 and 2000 and 2000-2015: Belarus, Botswana, Kazakhstan, Korea DPR, Mongolia, Netherlands, Rwanda, Singapore, St Vincent and Grenadines, Suriname, and Turkey.

The second pathway is to calculate the rates of decline required for each country to achieve a maternal mortality ratio of no more than 70/100 000 live births by 2030. Since 111 countries are already on track to achieve a ratio lower than that threshold, this scenario leads to an aggregate global maternal mortality ratio of 52/100 000 live births by 2030. But it would require 20 countries to achieve average annual declines greater than 12.2%, the fastest rate registered over 2005-15, by Kazakhstan.

**Fig 3:**
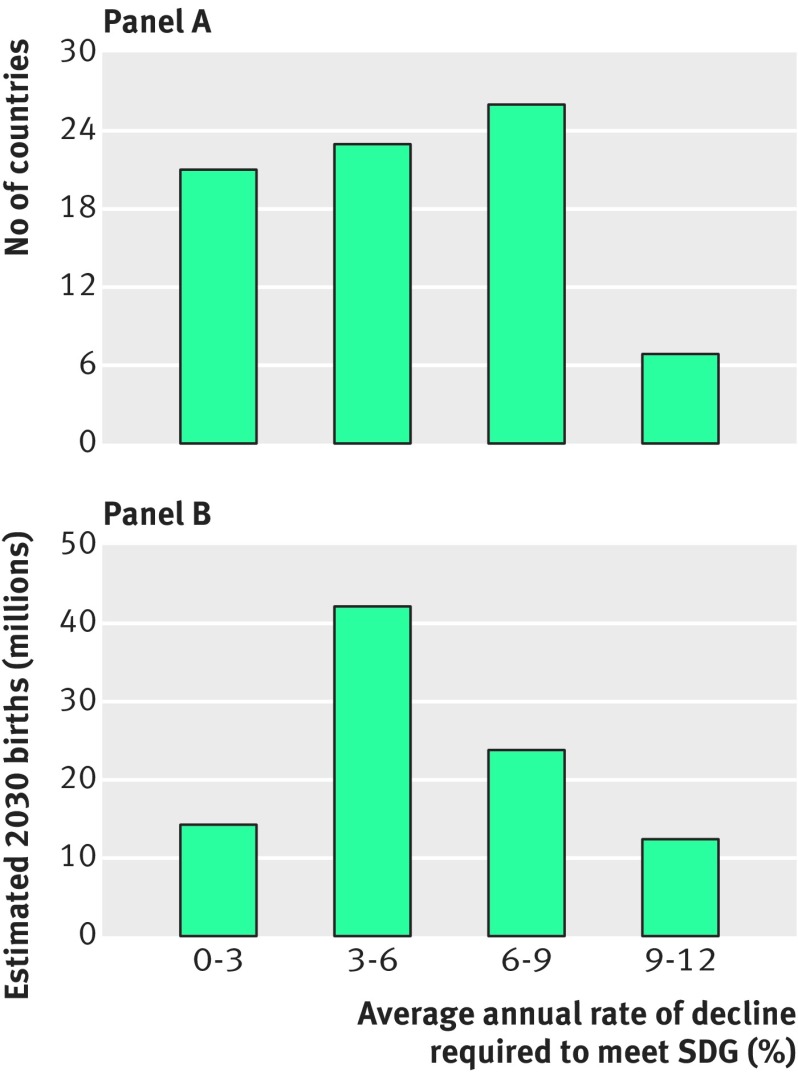
Distribution of average annual rates of fall required to meet 2030 sustainable development goal for child mortality among countries that had not met target in 2016, by country and by estimated number of births in 2030 for those countries^1^ ^6^

Child mortality is more straightforward to analyse because the SDG target is set at country level. Seventy seven countries had not already achieved the SDG standard in 2016, and these countries experienced an unweighted average annual fall in child mortality of 3.8% during 2006-16. Figure 3 shows the annual rates of decline required for these countries to meet the 2030 target. Twenty one countries—with 14 million births projected in 2030—require average annual reductions of 0-3% over 14 years. Meanwhile 23 countries (with 42 million births) require annual reductions of 3-6%; 26 countries (with 24 million births) require annual reductions of 6-9%; and seven (with 12 million births) require annual reductions of 9-12%. For comparison, Kazakhstan and Rwanda recorded the fastest annual rates of reduction during 2006-16, at 9.3% and 9.2%, respectively. Somalia had the highest estimated child mortality in 2016, at 133 deaths per 1000 live births, and thus requires the fastest rate of decline to meet the 2030 target—an annual rate of 11.1%; this compares with the 2.7% it achieved from 2006 to 2016.

## Lives at stake

For countries currently off track on either of the mortality targets, we estimated the approximate number of “lives at stake” under business as usual trends. We calculated this by estimating the cumulative difference between the number of deaths over the 14 years from 2017 through 2030 under current trajectories and the number of deaths over the same period if the maternal and child mortality targets are achieved. 

Table 1 presents summary results for maternal mortality if each country achieves a maternal mortality of no more than 70 per 100 000 live births (the second scenario described earlier). About 1.6 million mothers’ lives are at stake through to 2030. Of these, 1.1 million (67%) are at stake in 10 countries. Nigeria accounts for one third of the total and needs to accelerate its annual rate of decline in maternal mortality from 1.5% during 2005-15 to an extraordinarily rapid 15.1% during 2015-30.

**Table 1:** Estimated number of maternal lives at stake if maternal mortality ratio of 70/100 000 live births is not met in all countries by 2030, cumulative 2017-30^2^ ^6^ (10 countries with largest gap listed separately)

Country	No of deaths on current trend (000s)	No of deaths if target met (000s)	No of lives saved (000s)
Nigeria	812	274	539
Democratic Republic of Congo	336	118	218
Ivory Coast	80	29	50
Niger	79	34	45
Kenya	88	43	45
Malawi	67	22	44
Chad	66	25	41
Cameroon	66	27	39
India	408	369	39
Somalia	61	23	38
**Subtotal**	2061	964	**1097**
60 other off-track countries	1330	795	534
**Total**	3390	1759	**1631**

Table 2 shows the corresponding results if each country achieves a child mortality of no more than 25/1000 live births. Around 10.2 million additional children’s lives will be saved by 2030 if the SDG target is achieved, a total consistent with recent UN estimates.^1^ Our results draw further attention to the concentrated nature of the challenge, with 10 countries accounting for 8.3 million (81%) of the lives at stake. Just three countries—Nigeria, Pakistan, and Democratic Republic (DR) of Congo—account for more than 5.9 million (58%) of the lives at stake. Nigeria is the country with the most children’s lives at stake, at nearly 3.1 million. To achieve the target, its annual rate of fall in child mortality needs to accelerate from 3.7% during 2006-16 to 9.6% during 2016-30. Pakistan needs to see its rates of fall accelerate from 2.3% to 7.7% and DR Congo from 3.4% to 8.9% (see appendix on bmj.com for all country level calculations, including supplemental results for neonatal mortality).

**Table 2:** Estimated number of lives at stake among children under 5 years if target of 25/1000 live births is not met in all countries by 2030, cumulative 2017-2030^1^ ^6^ (10 countries with largest gap listed separately)

	No of deaths on current trends (000s)	No of deaths if target met (000s)	No of lives saved (000s)
Nigeria	8961	5903	3058
Pakistan	4979	3409	1570
Congo, Democratic Republic	3945	2644	1300
Somalia	1107	606	501
Chad	1044	581	463
Mali	1031	664	366
Ivory Coast	1017	673	344
Sudan	1078	821	257
Niger	1074	851	223
Benin	532	321	211
**Subtotal**	24 766	16 473	8294
38 other off-track countries	13 893	11 972	1921
**Total**	38 660	28 445	10 214

Three countries have data for child mortality but are missing total births: Dominica, Marshall Islands, and Nauru. Total number of countries off track for child mortality is 51.

## Strategies to accelerate progress

With so many lives at stake, accelerating the rates of progress in countries that are not on track to reach the SDG3 targets for maternal and child health is a critical global health priority. Given the differences between countries in characteristics such as health system capacity, fertility rate, and the main causes of death, there is no “one size fits all” approach to achieving such acceleration.^9^ Nevertheless, several health sector interventions—as well as policies outside the health sector—could promote acceleration across all low and middle income countries that are off-track.

### Health sector interventions

Most importantly, three integrated packages of essential health interventions need to be scaled up: a reproductive health package, a maternal and newborn health package, and a child health package.^10^ ^11^ The new edition of *Disease Control Priorities*, a compendium of evidence on the effectiveness of global health interventions, describes 61 interventions that should ideally be included in these three packages. Scaling up these interventions would be highly cost effective, with an estimated benefit:cost ratio of 7-11 for 2015-35.^11^

The countries listed in table 1 and table 2 typically have weak health delivery systems, and, as noted by the Lancet Commission on Investing in Health, expanding coverage of essential interventions will therefore require “structural investments in the health system” (eg, human resource, infrastructure, and supply chain investments).^12^ In particular, investments are needed to ensure that these packages can be delivered across a community platform (community workers or health posts) and in primary health centres and hospitals.

Each country’s tailored strategy needs to prioritise the interventions that will have the greatest effect on local causes of death. For example, in 2015, acute lower respiratory infection was the number one cause of child death in seven of the 10 countries with the highest mortality (Nigeria, DR Congo, Somalia, Chad, Sudan, Niger, Benin); malaria was the lead cause in two countries (Mali and Ivory Coast); and prematurity was the lead cause in Pakistan.^13^ For all these countries, progress on child mortality can be accelerated by scaling up the detection and treatment of childhood infections at the community, primary care, and hospital level, including community referral of children with danger signs. Other key interventions include management of severe acute malnutrition, expanding contraception, management of labour and delivery, and care of preterm births.

Expanding coverage of such health services will require dedicated financing, starting with domestic resources. External financing, especially for strengthening health systems, will also continue to be important for low and lower middle income countries, particularly those that are affected by conflict.^14^ Proactive international strategies will be needed to ensure continuity of health services in countries that are graduating from traditional aid thresholds. For example, over the coming decade Ivory Coast and Pakistan will surpass the current support threshold of Gavi, the Vaccine Alliance, if these countries maintain their average rate of real per capita economic growth from 2006 to 2016.

Other health systems factors have been linked with accelerated progress on child and maternal mortality.^9^ ^15^ These include strong leadership, taking action to improve the quality of care, reducing inequities in coverage, and developing “capacities to collect, analyse and use robust evidence to inform policy, investment, implementation and accountability.”^9^ Investing in discovery, development, and delivery of new health technologies will also be important.^12^ The recent Innovation Countdown 2030 study used a Delhi process including more than 60 global health experts, who ranked the most promising innovations. Twelve innovations were ranked the highest in terms of their potential effect on maternal and child health.^16^ These include new formulations of oxytocin and uterine balloon tamponade to control postpartum haemorrhage; new treatments for severe childhood diarrhoea; and low cost, reusable, and easy to use resuscitators to improve newborn survival in low resource settings.

### Policies outside health sector

Health outcomes are also driven by complementary advances outside the health sector, although estimates vary regarding their quantitative importance.^9^ ^17^ Examples of positive contributors include improving sanitation, expanding access to clean water, expanding girls’ education, and improving incomes. The UN’s Global Strategy for Women’s, Children’s and Adolescents’ Health (2016-2030) therefore charts a path that is “universal in scope and multisectoral in action.”^18^ Targeted policy efforts are also required to ensure relevant health and non-health interventions reach traditionally marginalised populations, consistent with a rights based approach that fulfils the SDG ambition that no one is “left behind.”

## Conclusion

Universal achievement of the SDG targets for maternal and child health requires accelerated progress across 79 countries. Overall, roughly 11.8 million lives can be saved if the targets are reached, including 1.6 million mothers and 10.2 million children. Close to seven million (57%) of the lives are at stake in only three countries: Nigeria, Pakistan, and DR Congo. To reach the SDG benchmarks for both maternal and child mortality, Nigeria will need to achieve faster average annual rates of decline than those of any countries recorded over the most recent decade. Considerable evidence exists regarding the interventions needed to achieve these standards. But success will not arise through business-as-usual approaches.

**Key messages**

42 countries are not on track to achieve the sustainable development goal targets for both maternal and child mortality
Another 37 countries will miss at least one of these thresholds
The lives of 1.6 million mothers and 10.2 million children will be saved if all countries meet the thresholds
The rates of decline required in countries with the biggest gaps are very high
Scaling up integrated packages of evidence based interventions, both inside and outside the health sector, will be essential to accelerate progress

## Web Extra

Extra material supplied by the author

Supplementary data on trends in maternal mortality ratio and child mortality rates

File: mcaj42175.ww1

## Notes

All MMR data up to 2015 are based on official World Bank country level and aggregate estimates as downloaded on 23 October 2017. Aggregate MMR estimates for 2016-2030 use country level birth weights based on UN Population Division, 2017, which imply slightly different global aggregate MMR estimates for 2015. The World Bank reports global MMR in 2015 as 216/100 000 live births. Our method using UN Population Division estimates implies a global MMR that year of 218/100 000 live births. Note also that UN Population Division birth data are reported for cumulative five year periods (eg, 2000-05, 2005-10). We estimate annual birth values by dividing each five year cumulative value by 5 and marking that as the nominal value for the third listed year of the period (eg, 2002, 2007). Intervening years are then interpolated based on five year compound annual growth rates. For under 5 child mortality, our methods produce a slightly higher baseline number of total deaths in 2016, at 5.7 million, compared to 5.6 million reported by the UN Interagency Group for Child Mortality Estimation. Statistical code is available from the corresponding author.

Contributors and sources: JWMcA, an economist, has studied and been active in policy deliberations relating to the millennium development goals (MDGs) and sustainable development goals (SDGs); KR has conducted research on the MDGs and SDGs; GY has studied and published widely on issues of global health, including as lead writer of the Global Health 2035 report. The article draws from official UN and World Bank data sources and arose from discussions at a conference session focused on global health trajectories. JWMcA co-led drafting and overall analysis. KR managed data analysis, created figures, and contributed to drafting. GY co-led drafting and overall analysis.
